# Supplementary material for: Laser-induced topological spin switching in a 2D van der Waals magnet
Source: Nat Commun. 2023 Mar 13;14:1378. doi: 10.1038/s41467-023-37082-y (PMC10011585; doi:10.1038/s41467-023-37082-y)
Supplement: Supplementary file 1 — Supplementary Information [file 41467_2023_37082_MOESM1_ESM.pdf]

Supplementary Information:
Laser-induced topological spin switching in a 2D van der Waals magnet

Maya Khela[1], Maciej Dąbrowski[2†], Safe Khan[3], Paul S. Keatley[2], Ivan Verzhbitskiy[4,5], Goki Eda[4,5], Robert J. Hicken[2], Hidekazu Kurebayashi[3,6], Elton J. G. Santos[1,7†]

[1] *Institute for Condensed Matter Physics and Complex Systems, School of Physics and Astronomy, The University of Edinburgh, EH9 3FD, United Kingdom*

[2] *Department of Physics and Astronomy, University of Exeter, EX4 4QL, United Kingdom*

[3] *London Centre for Nanotechnology, University College London, 17-19 Gordon Street, London, WCH1 0AH, UK*

[4] *Department of Physics, National University of Singapore, Singapore, Singapore*

[5] *Centre for Advanced 2D Materials and Graphene Research Centre, Singapore, Singapore*

[6] *Department of Electronic & Electrical Engineering, UCL, London WC1E 7JE, United Kingdom*

[7] *Higgs Centre for Theoretical Physics, The University of Edinburgh, EH9 3FD, United Kingdom*

[†] *Corresponding author: M.K.Dabrowski@exeter.ac.uk, esantos@ed.ac.uk*

Supplementary Movies

Supplementary movies are included using the following description:

- Supplementary Movie S1: Zero-field cooling spin dynamics with the magnetisation projected along (left) M_x and (right) M_z components. All interactions included in Eq. 1 are considered except DMI which is set to zero ($|\boldsymbol{A}_{ij}| = 0$).
- Supplementary Movie S2: Spin dynamics along the (left) in-plane M_x and (right) out-of-plane M_z components of the magnetisation after laser excitation at 0.03 mJ cm^{-2} on $CrGeTe_3$.
- Supplementary Movie S3: Similar as movie S1 but at a fluence of 0.06 mJ cm^{-2}.
- Supplementary Movie S4: Similar as movie S1 but at a fluence of 0.14 mJ cm^{-2}.
- Supplementary Movie S5: Similar as movie S1 but at a fluence of 0.20 mJ cm^{-2}.
- Supplementary Movie S6: Similar as movie S1 but at a fluence of 0.30 mJ cm^{-2}.
- Supplementary Movie S7: WFKM measurements (as in Supplementary Fig.4) with the out-of-plane magnetic field applied by an air-core electromagnetic coil. The movie shows the evolution of the domain structure within the field range -74 mT to +36 mT. The images in the movie are 25 $\times$ 25 mm.
- Supplementary Movie S8: The domain structure evolution during the magnetization reversal from –H (-250 mT) to +H (+250 mT) (red circles in Supplementary Fig.5).

		$B_{dip} \neq 0$		$B_{dip} = 0$	
		$K_{ij} \neq 0$	$K_{ij} = 0$	$K_{ij} \neq 0$	$K_{ij} = 0$
DMI	Applied Field	**Skyrmions**<br>**Skyrmionium**	**Skyrmions**<br>**Skyrmionium**	**Skyrmions**<br>**Skyrmionium**	**Skyrmions**
	No Applied Field	**Skyrmions**	**Skyrmions**	**Skyrmions**<br>**Skyrmionium**	**Skyrmions**

Supplementary Table 1: Effect of the inclusion of different interactions and an applied field on the formation of non-trivial spin structures (skyrmions, skyrmionium) in $CrGeTe_3$ via cooling simulations. Skyrmions and anti-skyrmions are formed simultaneously even though just skyrmions are mentioned in the table for simplicity. Biquadratic exchange (K_{ij}), dipole field (B_{dip}) and Dzyaloshinskii-Moriya interactions (DMI) and an external out of plane magnetic field (11.7 mT) are considered. Simulations without any DMI resulted in trivial magnetic stripe domains and bubbles without any topological features. The inclusion of DMI in $CrGeTe_3$ follow that in Ref.[1].

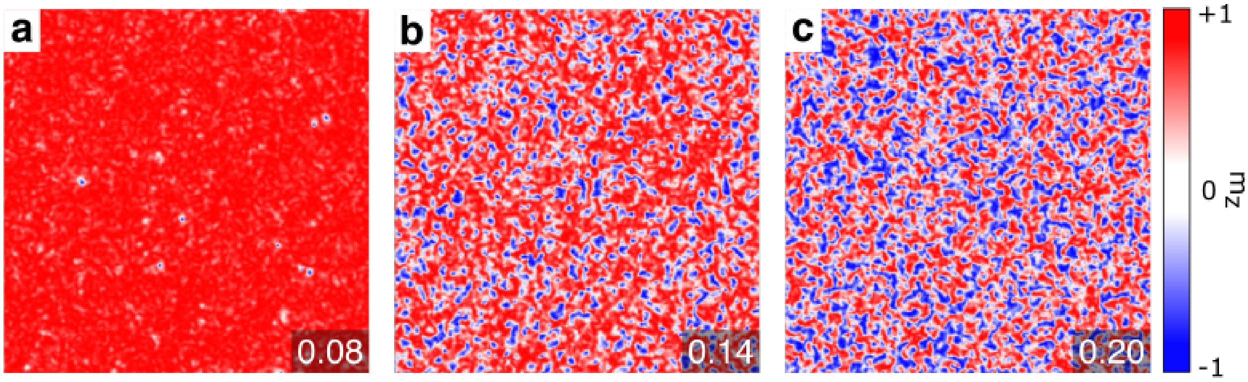

Supplementary Figure 1: **Magnon droplets. a-c,** Snapshots of the out-of-plane magnetisation M_z of $CrGeTe_3$ subjected to laser pulses of different fluences at 0.08 mJ cm^{-2}, 0.14 mJ cm^{-2} and 0.20 mJ cm^{-2}, respectively. Each panel was extracted at $t = 115$ ps (15 ps after the pulse). This is the approximate end of the period of magnon localisation. At low fluence (0.08 mJ cm^{-2}) as in **a** very few magnon droplets are nucleated, and thus there is little opportunity for skyrmion formation. As fluence increases (0.14 mJ cm^{-2} in **b**) many magnon droplets are formed, and they remain isolated as they gain topological protection rather than merging. This provides the opportunity for dense skyrmion formation. At high fluence intensities (0.20 mJ cm^{-2} in **c**) several magnon droplets are nucleated, but thermal fluctuations inhibit their stabilisation. As a result many magnon droplets merge into labyrinthine domains with some still keeping skyrmion characteristics.

Fluence (mJ cm^{-2})	Equilibration Temperature (K)
0.03	0.34
0.05	0.56
0.08	0.90
0.10	1.12
0.12	1.35
0.14	1.57
0.16	1.80
0.18	2.02
0.20	2.25
0.30	3.37

Supplementary Table 2: Calculated equilibration temperatures (K) for the system after laser excitation at different fluences (mJ cm^{-2}) on $CrGeTe_3$. Values are extracted of Figure 1 after the system reached equilibrium.

Symbol	Name	Value
C_{e0}	Electron heat capacity constant	736.87 $Jm^{-3}K^{-2}$
C_p	Phonon specific heat capacity	8.90×10^6 $Jm^{-3}K^{-1}$
G	Electron-phonon coupling factor	5.52×10^{16} $Jm^{-3}K^{-1}s^{-1}$

Supplementary Table 3: Calculated material parameters for the 2TM used on the ultrafast laser-induced magnetic dynamics on $CrGeTe_3$.

Topological spin switching: simulations

To investigate the toggle switch found in the measurements (Figure 3), we simulate the variation of the topological spin textures with laser excitations also taken into account an external magnetic field. We considered similar steps as those used in the WFKM measurements: under a field of +25 mT (Fig. 2**a-c**), 0 mT (Fig. 2**d-f**) and -25 mT (Fig. 2**g-i**). We quantified the effect in terms of the topological number N_{sk} since the coexistence between skyrmions and stripe domains over the surface complicates their clear identification by visualisation methods. N_{sk} will provide a quantitative metric of the amount of changes between the spin textures involved without adding more complex elements to the analysis. We started with a configuration with $N_{sk} = -1.7$ (Fig. 2**b**) which was previously obtained from a saturated FM state under a single laser pulse. This state then is further equilibrated under a field of +25 mT and two additional laser pulses applied. We observed a substantial increment in the amount of skyrmions and sign reversal of the spin textures ($\mathrm{N}_{sk} = +16.1$) after several nanoseconds of thermalisation (Fig. 2**c**). The process is repeated under zero field (Fig. 2**d**) taking the previous equilibrated spin state (Fig. 2**c**) as the reference (Fig. 2**e**) on the new switching step. Interestingly, the additional laser pulse at 0 mT reduces the number of skyrmions in the layer (Fig. 2**f**) but keeps the same sign ($N_{sk} = +5.5$) as in the previous state. We repeat this process at a different field polarity (-25 mT, Fig. 2**g-i**) to recover a spin configuration relatively close to the original state (Fig. 2**b**) but slightly smaller topological number ($N_{sk} = -6.1$). The simulations shown in Fig.2 reproduced at least qualitatively the experimental observations in Figure 3, and provided a theoretical background for further exploration. It is worth mentioning that each ultrafast spin dynamics simulation for each laser pulse and magnetic field is

very time-intensive ($\sim 24-36$) hours requiring a large load of computational resources. Indeed, the simulation of $N = 100$ pulses is unpractical due to their large computational cost.

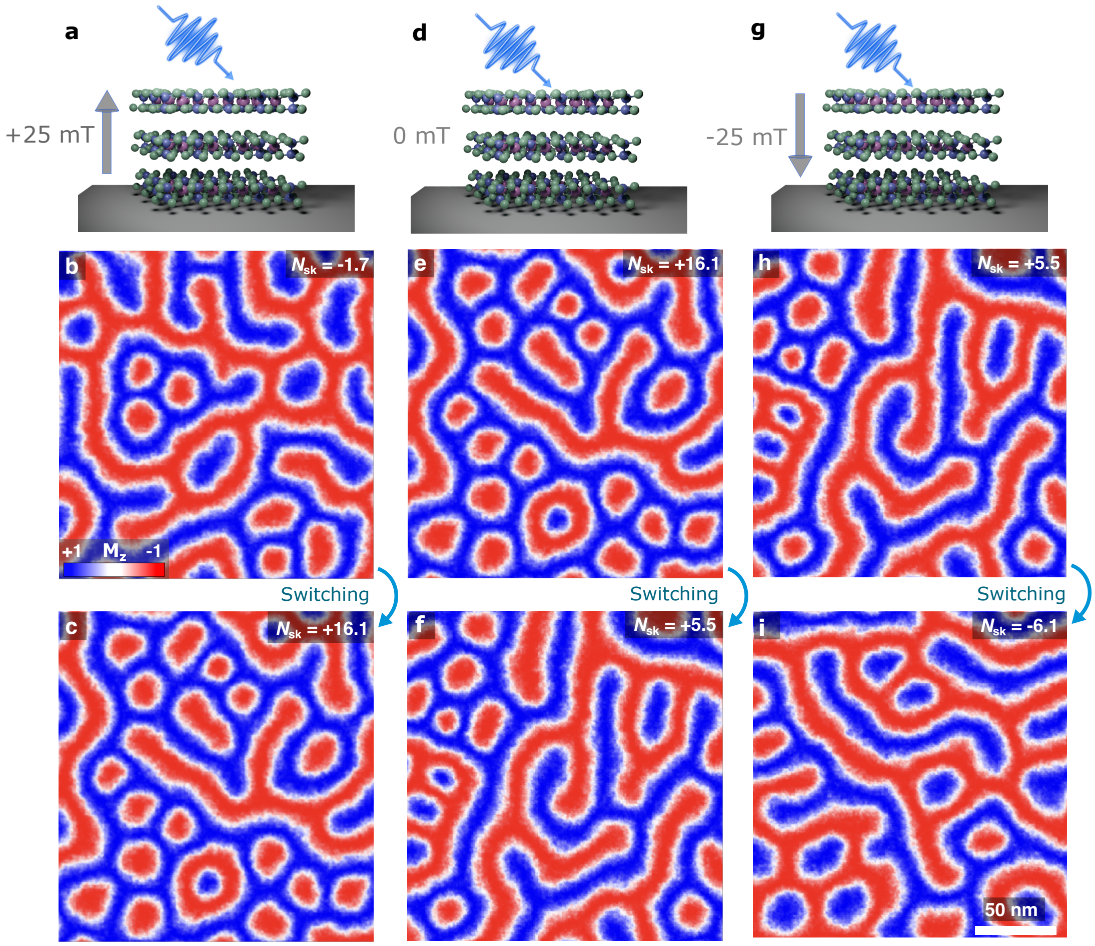

Supplementary Figure 2: **Topological spin switching: simulations.** **a,** Schematic of $CrGeTe_3$ layers under laser excitations and an applied out-of-plane magnetic field (+25 mT) which will be used in the ultrafast dynamics in **b** and **c**. **b-c,** Snapshots of the out-of-plane magnetisation (M_z) at 1.6 K showing a magnetic state with a skyrmion number of $N_{sk} = -1.7$ and $N_{sk} = +16.1$, respectively. The former state is characterised by a large amount of spirals whereas the latter has an increased number of skyrmions quasiparticles induced by the laser. A spin switching occurred between both states as two laser pulses of 0.18 mJ cm^{-2} are applied to the system under a magnetic field of +25 mT. The initial state ($N_{sk} = -1.7$) is obtained from a fully saturated magnetised state ($|M_z| = 1$ and $N_{sk} = 0$). **d,** Similar as **a** but without any applied field. **e,** Once the state in **c** is stabilised, it is used as an initial reference under the laser excitations to generate a new magnetic configuration with $N_{sk} = +5.5$ as shown in **f**. No magnetic field is applied during this process. **g,** Similar as in **a** but with a magnetic field of opposite polarity (-25 mT). **h,** This reference configuration corresponds to the stated created in **f** which will be under one laser pulse and a field of -25 mT to generate the spin configuration in **i**.

Sample characterization

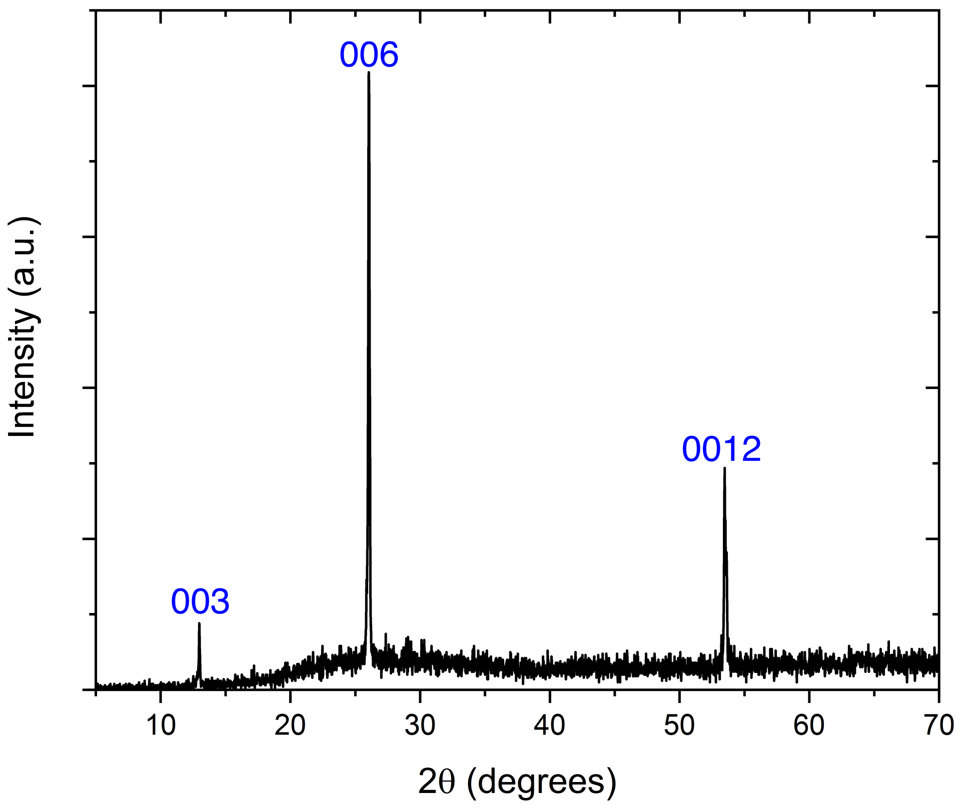

Supplementary Figure 3: **X-ray characterisation for $CrGeTe_3$ crystals.** X-ray diffraction patterns of pristine bulk crystal of $CrGeTe_3$ used in the measurements. The plot shows a series of out of plane (00l) peaks which agrees well with previous results in the literature[2].

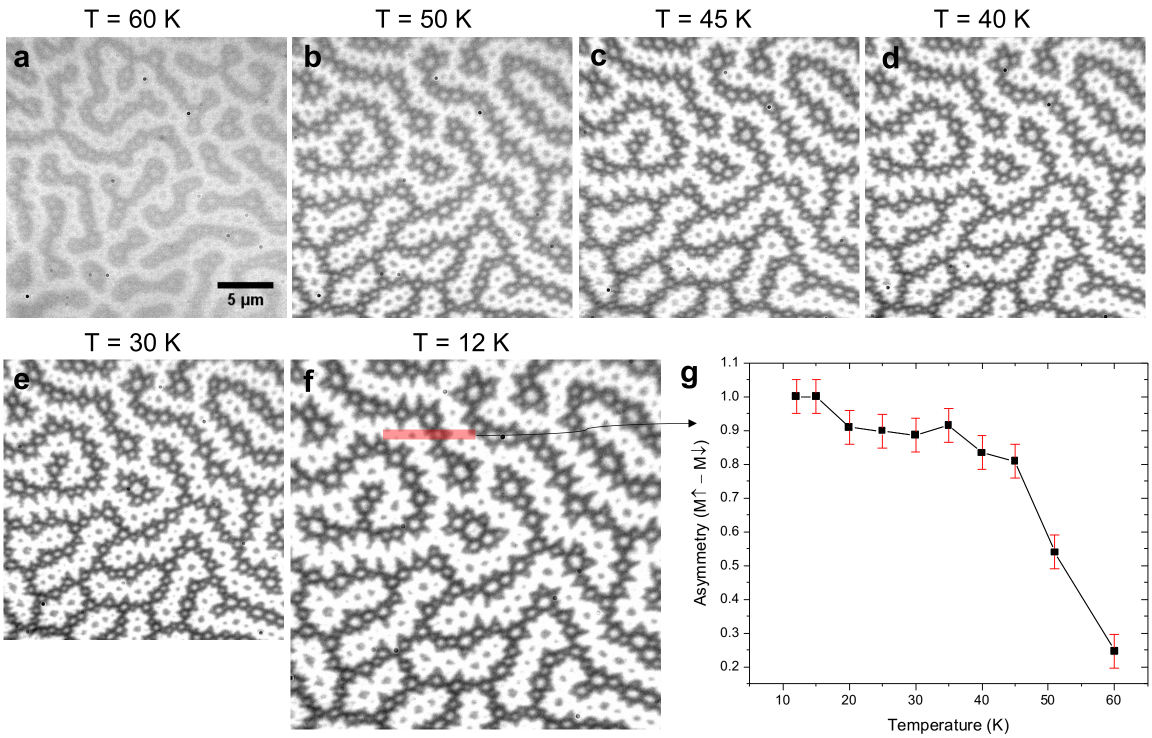

Supplementary Figure 4: **Temperature dependence of the domain structure and Kerr signal in CrGeTe$_3$. a-f,** Domain structure images were performed by wide-field Kerr microscopy (WFKM) (as described in Methods) in a polar geometry sensitive to the out-of-plane magnetization. The sample illumination was linearly polarized LED white light, while polarization changes of the reflected light due to the polar Kerr effect were detected as intensity changes using a nearly crossed analyzer and quarter-waveplate. The measurements were performed at remanence and within the temperature range 65−12 K. **g,** Variation on the polarisation for spin up and down as a function of the temperature at the area highlighted in **f**.

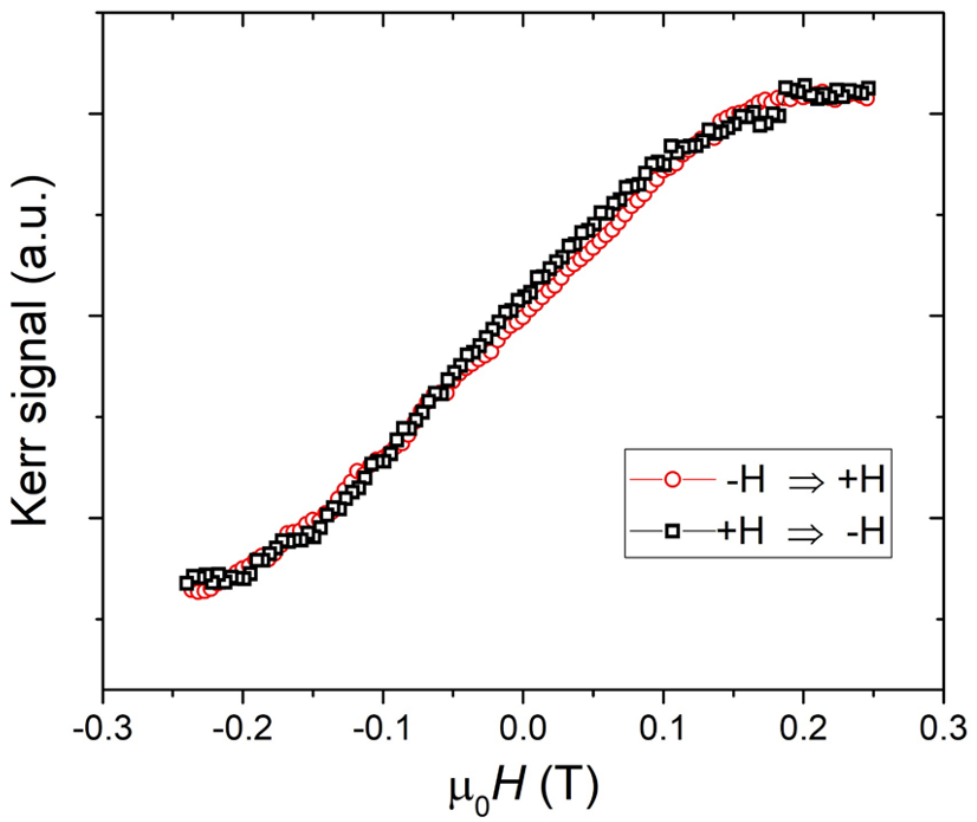

Supplementary Figure 5: **Hysteresis loop for $CrGeTe_3$ crystals.** The hysteresis loop extracted from domain structure images acquired with beam-scanning Kerr microscopy using microstat MO 5T superconducting magnet, with the field direction applied perpendicular to the sample surface. The sample illumination was linearly polarized CW green laser diode, while polarization changes of the reflected light due to the polar Kerr effect were detected using a balanced polarizing photodiode bridge detector. The measurements were performed at 6 K. The domain structure evolution during the magnetization reversal from –H (-250 mT) to +H (+250 mT) (red circles) can be observed in the Supplementary Movie S8.

Supplementary references

1. Zhang, Y. *et al.* Emergence of skyrmionium in a two-dimensional crge$(\mathrm{se},\mathrm{te})_3$ janus monolayer. *Phys. Rev. B* **102**, 241107 (2020). URL `https://link.aps.org/doi/10.1103/PhysRevB.102.241107`.

2. Khan, S. *et al.* Spin dynamics study in layered van der waals single-crystal $cr_2ge_2te_6$. *Phys. Rev. B* **100**, 134437 (2019).
