# Supplementary material for: Laser-induced topological spin switching in a 2D van der Waals magnet
Source: Nat Commun. 2023 Mar 13;14:1378. doi: 10.1038/s41467-023-37082-y (PMC10011585; doi:10.1038/s41467-023-37082-y)
Supplement: Supplementary file 3 — Description of Additional Supplementary Files [file 41467_2023_37082_MOESM3_ESM.pdf]

Title: Supplementary Movie 1
Description: Zero-field cooling spin dynamics with the magnetisation pro-jected along (left) M_x and (right) M_z components. All interactions included in Eq. 1 are considered except DMI which is set to zero ($|A_{ij}| = 0$).

Title: Supplementary Movie 2
Description: Spin dynamics along the (left) in-plane M_x and (right) out-of-plane M_z components of the magnetisation after laser excitation at 0.03 mJ cm^{-2} on $CrGeTe_3$.

Title: Supplementary Movie 3
Description: Similar as movie S1 but at a fluence of 0.06 mJ cm^{-2}.

Title: Supplementary Movie 4
Description: Similar as movie S1 but at a fluence of 0.14 mJ cm^{-2}.

Title: Supplementary Movie 5
Description: Similar as movie S1 but at a fluence of 0.20 mJ cm^{-2}.

Title: Supplementary Movie 6
Description: Similar as movie S1 but at a fluence of 0.30 mJ cm^{-2}.

Title: Supplementary Movie 7
Description: WFKM measurements (as in Supplementary Fig.4) with the out-of-plane magnetic field applied by an air-core electromagnetic coil. The movie shows the evolution of the domain structure within the field range -74 mT to +36 mT. The images in the movie are 25 × 25 mm.

Title: Supplementary Movie 8:
Description: The domain structure evolution during the magnetization reversal from –H (-250 mT) to +H (+250 mT) (red circles in Supplementary Fig.5).
